# Theanine modulation of monoamine metabolism visualized by derivatized imaging mass spectrometry

**DOI:** 10.1038/s41598-025-08190-0

**Published:** 2025-07-02

**Authors:** Shu Taira, Aya Abe, Makoto Ozeki, Hitomi Shikano, Mahendra P. Kapoor, Makoto Muto, Shoko Kobayashi

**Affiliations:** 1 Faculty of Food and Agricultural Sciences, Fukushima University, Kanayagawa, Fukushima, 960-1296 Japan; 2 Nutrition Division, Taiyo Kagaku Co. Ltd., Yokkaichi, Mie 510-0844 Japan; 3 Faculty of Fisheries Sciences, Hokkaido University, 3-1-1 Minato, Hakodate, Hokkaido 04108611 Japan

**Keywords:** Analytical biochemistry, Imaging, Neurochemistry, Health care

## Abstract

L-Theanine (LT), a derivative of glutamic acid, is abundant in tea leaves and contributes to tea’s umami and sweetness. In recent years, its neurological effects, notably its role in promoting relaxation, have attracted intense research interest. The purpose of this study was to elucidate the detailed mechanisms underlying the relaxation effects of LT by thoroughly investigating the dynamics of neurotransmitters in the brain using mice after LT administration. Imaging mass spectrometry (IMS) was employed to visualize changes in the catecholamine system within the brain after LT administration. Simultaneous imaging of catecholamines, such as L-dihydroxyphenylalanine, dopamine, and norepinephrine, as well as representative amino acids such as γ-aminobutyric acid, revealed dynamic variations across time. Our findings revealed links between LT administration and the modulation of both inhibitory and excitatory neurotransmission, through its influence on the GABAergic and catecholaminergic systems, following the entry of exogenous LT into the brain. This approach enhanced our understanding of the mechanisms underlying LT’s effects on the brain by visualizing neurotransmitter and amino acid distribution, as well as accompanying metabolic processes, at specific time points using IMS.

**Supplementary Information:**

The online version contains supplementary material available at 10.1038/s41598-025-08190-0.

## Introduction

L-theanine (LT; γ-glutamylethylamide), a non-protein amino acid isolated from green tea, has numerous health benefits, including relaxation, stress reduction^1,2^, antihypertensive effects^3,4^, improved sleep quality^5^ and alleviation of schizophrenia symptoms^6^. Animal studies have demonstrated synergistic and antagonistic effects among LT and other bioactive compounds such as caffeine and catechins^7,8^. LT is absorbed in the small intestine in a concentration-dependent manner^9^ and it can cross the blood–brain barrier (BBB) via the amino acid leucine-preferring transport system^10^. Neurotransmitters are known to play a crucial role in modulating numerous types of signal transduction, including cognition, function, sleep, and emotion. LT has been demonstrated to influence the expression of brain-derived neurotrophic factor (BDNF)^11^ and modulate neurotransmitters such as L-dihydroxyphenylalanine (L-DOPA), dopamine (DA), norepinephrine (NE), serotonin (5-HT)^12,13^, and γ-aminobutyric acid (GABA). It has been postulated that LT’s fundamental mechanism involves antagonistic inhibition of glutamine transporters^14^ and glutamate receptors^15^.

LT has been reported to inhibit glutamine transport in neurons and astroglia potentially by competing with glutamine due to its structural similarity. This inhibition may limit glutamine conversion to glutamate in presynaptic neurons, thereby preventing neuronal cell death caused by excitotoxicity^14^. In addition, Kakuda et al. have reported that LT binds to glutamate receptor subtypes (AMPA, kinate and NMDA receptor) and blocks the binding of L-glutamic acid to the glutamate receptors in cortisol neurons^15^. The balance between excitatory and inhibitory impulses in neurons may shift, resulting in relaxing effects through the suppression of glutamate supply. Despite substantial investigation into the mechanisms underlying LT’s neurological effects, no studies have visually demonstrated the relationship between LT and the dynamics of catecholamines and amino acids in the brain. Visualizing these dynamical patterns may provide deeper insights into the pathways by which LT performs its relaxing and neuroprotective effects. Imaging mass spectrometry (IMS) recognizes and visualizes spatial information, allowing for the simultaneous detection of multiple analytes without the requirement for target-specific markers, such as antibodies^16–18^, in a single experiment. IMS has been widely employed across diverse fields, including biology^19^, pharmacology^18,20^, food chemistry^21–23^, plant science^24,25^, and neuroscience^26–28^. We previously demonstrated that derivatization reagents with a pyrylium core structure are effective for the detection of catecholamines and amino acids containing primary amine groups. Pyrylium salts interact with nucleophiles under alkaline scenarios, facilitating this reaction^29,30^. Among these chemicals, 2,4,6-trimethylpyrylium tetrafluoroborate (TMPy) efficiently reacts with monoamines amino groups. In this study, TMPy-based derivatized IMS was employed to evaluate amino acid and monoamine metabolism in the brain after LT administration. The objective was to identify the specific brain regions where these chemicals exhibit their effects and to trace their pathways using TMPy-based derivatized IMS, thereby revealing how they reach their target sites.

## Results and discussion

### Behavioral analysis

**Fig. 1:**
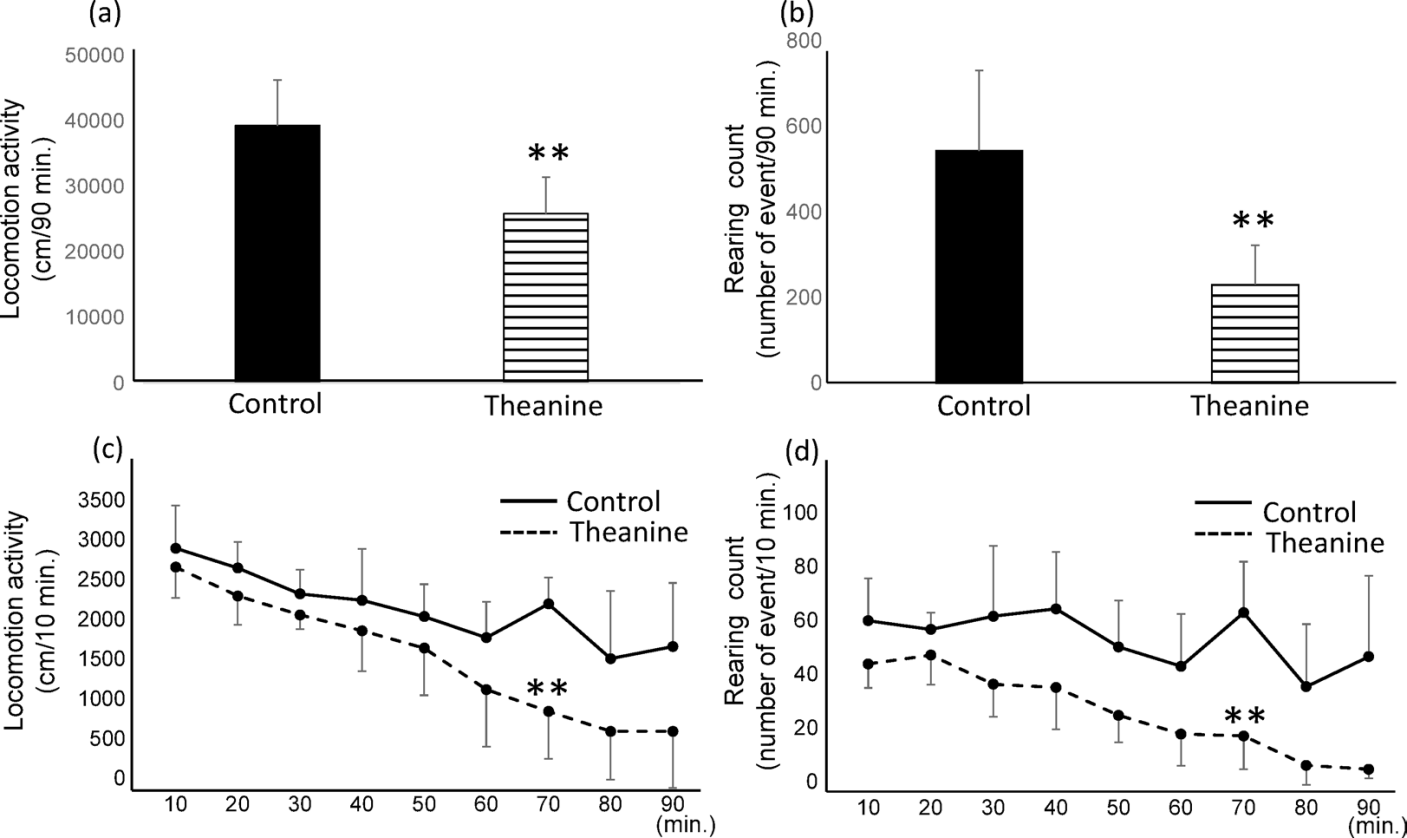
Comparison between control (n = 4) and theanine-administered mice (n = 5) for locomotor activity (**a** and **c**) and rearing (**b** and **d**). The values are expressed as mean ± SEM. *p < 0.05 and **p < 0.01 with Welch’s t-test. False Discovery Rate (FDR) correction was applied.

An open-field test was employed to evaluate the effect of LT administration on spontaneous movement. The ambulation (Fig. 1a) and rearing (Fig. 1b) behaviors of LT-administered mice were significantly reduced throughout 90 min compared to the control group. A time-based analysis indicated substantial differences between the control and the LT-administered groups, with statically significant reductions emerging 70 min. Although statical significance was not sustained at 80 and 90 min after multiple-comparison correction, the overall downward trend persisted across time, suggesting a sustained reduction in exploratory behave (Fig. 1c,d). Typically, mice exhibit ambulation and rearing as an exploratory behavior in novel environments. The observed decrease in behavioral activity in the LT-administered group suggested decreased stress and enhanced relaxation. To further examine the sedative effect of LT, we measured and visualized amino acid and catecholamine levels in the mouse brain using High performance liquid chromatography (HPLC)-electrochemical detector (ECD) and IMS, to unravel the underlying mechanisms of LT’s tension-relieving properties.

### HPLC-ECD analysis of catecholamines in LT-administered mice brain

**Fig. 2:**
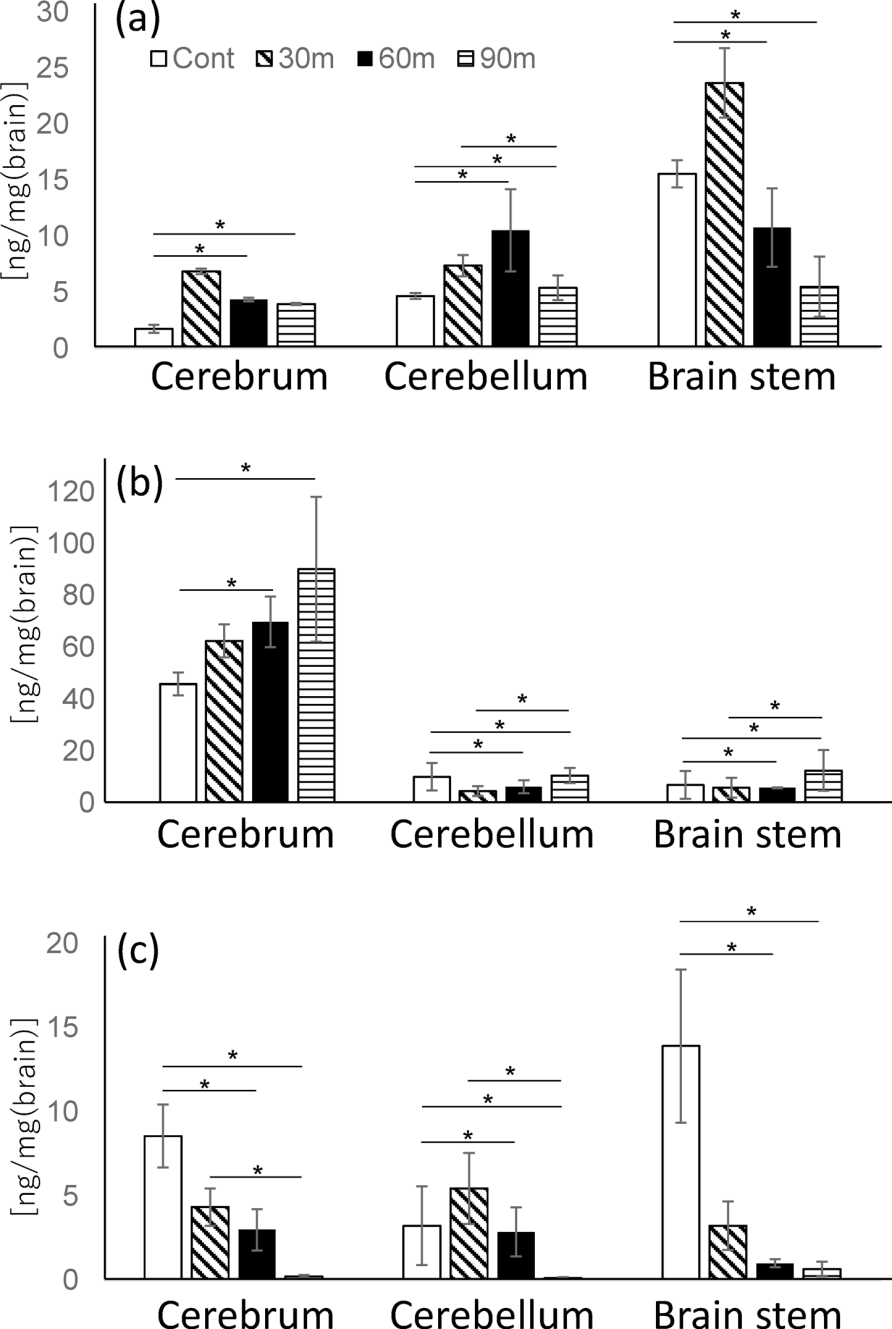
Quantitative comparison of the amount of L-DOPA (**a**) DA (**b**) and NE (**c**) in control group and post-theanine administration at 30, 60, and 90 min, respectively. The values are expressed as mean ± SEM. *p < 0.05 with Welch’s t-test. False Discovery Rate (FDR) correction was applied. n = 3.

The brain was divided into three distinct regions: the cerebrum, cerebellum (CB), and brainstem (BS), from which L-DOPA, dopamine (DA), and norepinephrine (NE) were extracted as described in the Experimental Section. Standards for these monoamines were validated under identical gradient conditions. The experiment revealed three distinct peaks corresponding to L-DOPA (retention time [RT]: 7.5 min), DA (RT: 11.7 min), and NE (RT: 4.2 min), respectively. These retention times are consistent with the principles of reversed-phase liquid chromatography, and represent the hydrophobic properties of the molecules. Monoamine concentrations were quantified using a standard curve based on peak area measurements. The concentrations of L-DOPA (Fig. 2a) in the cerebrum at control and at 30, 60, and 90 min were 1.7 ± 0.34, 6.8 ± 0.25, 4.3 ± 0.16, and 4.0 ± 0.1 ng/mg, respectively. Concentrations in the CB were 4.7 ± 0.25, 7.4 ± 0.95, 10.5 ± 3.7, and 5.4 ± 1.1 ng/mg, whereas in the BS, they were 15.5 ± 1.2, 23.6 ± 3.1, 10.8 ± 3.5, and 5.5 ± 2.6 ng/mg, respectively. A prior study found that L-DOPA is predominantly localized in the BS^31^, which is consistent with the present findings. L-DOPA levels increased after 30 min of LT administration compared to the control, followed by a steady drop at 60 and 90 min in the BS. The concentrations of DA in the cerebrum at control and at 30, 60, and 90 min were 45.8 ± 4.3, 62.4 ± 6.3, 69.9 ± 9.7, and 90.1 ± 27.9 ng/mg, respectively (Fig. 2b). Concentrations in the CB were 10.1 ± 5.2, 4.6 ± 1.9, 6.2 ± 2.4, and 10.5 ± 2.9 ng/mg, whereas in the BS, they were 6.9 ± 5.3, 5.9 ± 3.8, 5.8 ± 0.2, and 12.5 ± 7.8 ng/mg, respectively. DA is known to accumulate predominantly in the striatum (Str)^27,31,32^. The results revealed that DA was largely localized in the cerebrum, including the striatal region. In comparison to control group, DA levels increased 30 min after the LT administration. Subsequently, DA levels continued to rise over time significantly. Given that L-DOPA is a precursor to DA, the observed drop in L-DOPA levels, followed by a corresponding increase in DA levels over time, is consistent with established biochemical pathways and associated molecular mechanisms. The concentrations of NE in the cerebrum at control, 30, 60, and 90 min were 8.6 ± 1.9, 4.3 ± 1.1, 3.0 ± 1.2, and 0.2 ± 0.1 ng/mg, respectively (Fig. 2c). Concentrations in the CB were 3.2 ± 2.3, 5.4 ± 2.1, 2.9 ± 1.4, and 0.1 ± 0.07 ng/mg, whereas in the BS, they were 14 ± 4.5, 3.2 ± 1.4, 1.0 ± 0.2, and 0.7 ± 0.4 ng/mg, respectively. NE levels in the cerebrum and BS were considerably lower in the LT-administered group after 30 min compared to the control group. NE is released from the BS structure known as the locus coeruleus (LC) and primarily functions as a neurotransmitter in the cortex. It plays a critical role in regulating arousal, attention, stress responses, and cognitive processes such as learning and memory. The significantly lower NE levels in the cerebrum, cerebellum and BS of the LT-administered group are consistent with the decreased activity observed in behavioral assessments following LT administration.

### Confirmation of structure of TMPy-labelled amino acids and monoamines by MALDI tandem MS (MS/MS)

**Fig. 3:**
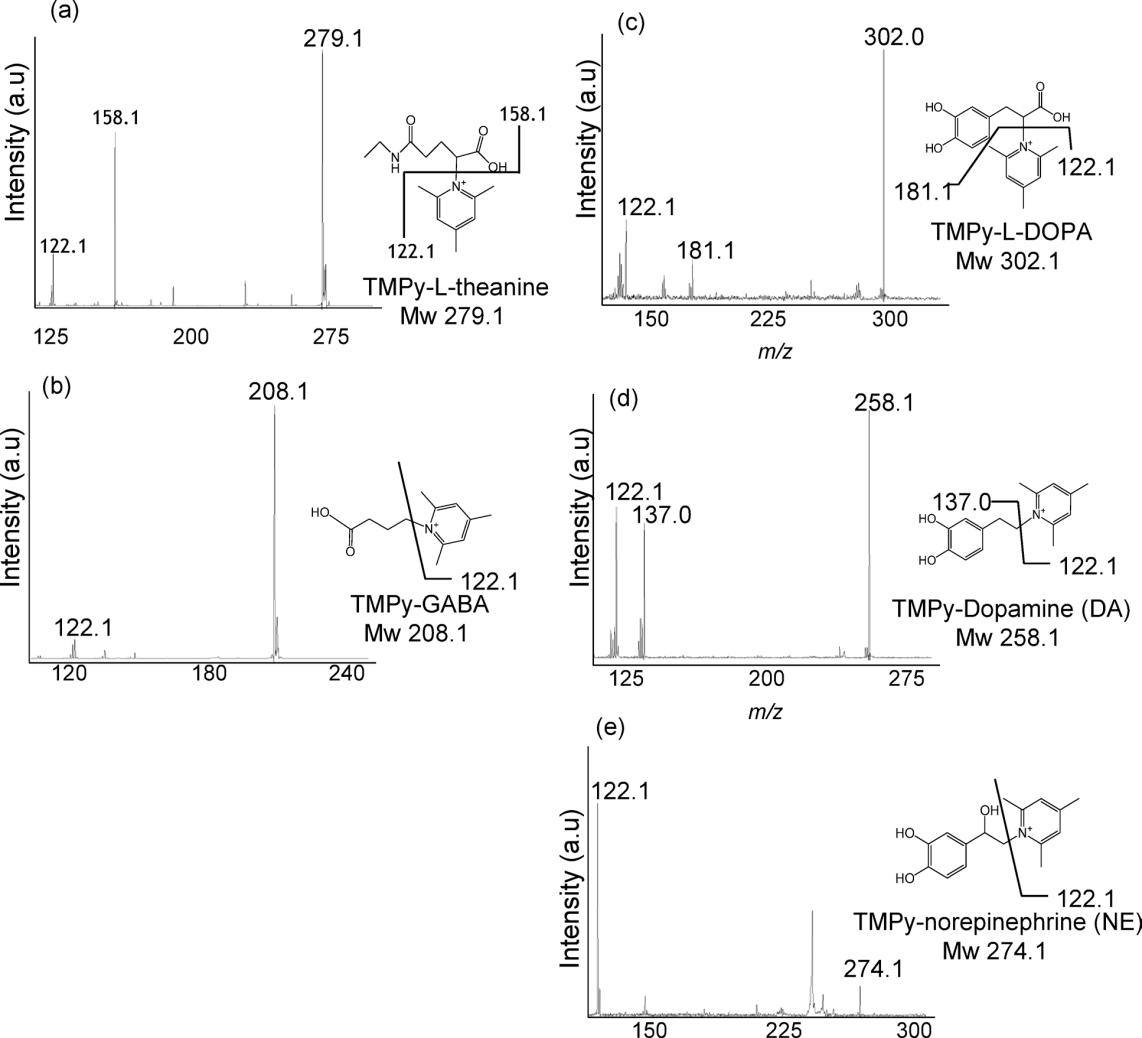
MS/MS spectra were acquired from mouse brain tissue sections. Theanine (**a**) was detected in the lateral ventricle (LV), GABA (**b**) in the cerebral cortex, L-DOPA (**c**) in the brainstem (BS), DA (**d**) in the striatum (Str), and norepinephrine (**e**) in the locus coeruleus (LC).

Previous study has demonstrated the improvement of ionization efficiency via the derivatization of TMPy^31^. We confirmed the derivatization of TMPy and the presence of target molecules in brain sections by MS/MS measurement (Fig. 3). All analyses were performed six times to ensure the reproducibility of the technique. The detected masses of TMPy-labelled LT (*m/z* 279.1: Fig. 3a), GABA (*m/z* 208.1: Fig. 3b), L-DOPA (*m/z* 302.1: Fig. 3c), DA (*m/z* 258.1: Fig. 3d) and NE (*m/z* 274.1: Fig. 3e) increased 105.0 Da compared with original mass (Mw 174.1, 103.1, 197.1, 153.1 and 169.1). A fragmented ion corresponding to the pyridine ring (*m/z* 122.1) was consistently cleaved and observed for all TMPy-modified target molecules. These results were consistent with the TMPy derivatization observed in each standard, demonstrating the reaction’s specificity. The detection of both the precursor ion and the fragmented TMPy ion on section unequivocally confirms that TMPy has modified the target molecule.

### LT distribution in the mouse brain using IMS

**Fig. 4:**
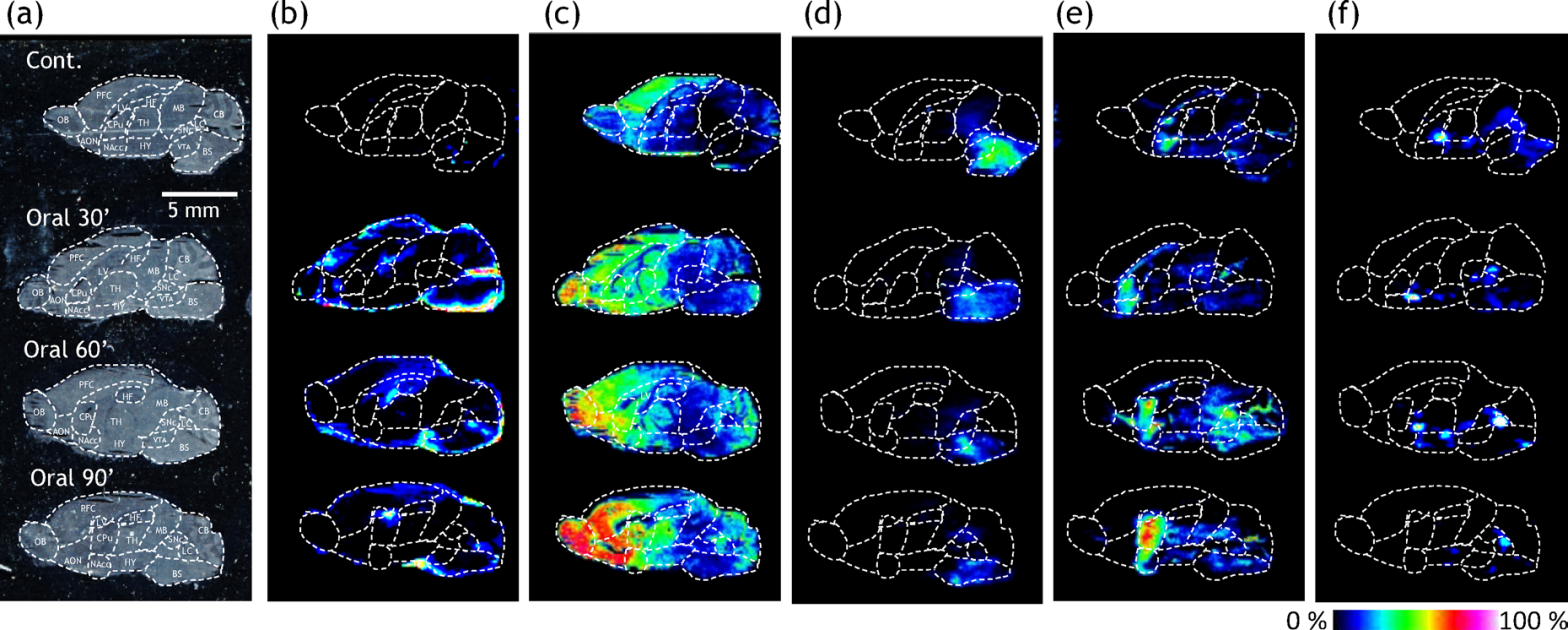
Derivatized imaging mass spectrometry of amino acids and catechol amines. Optical image of sagittal section from mouse brain (**a**). MS spectra reconstructed image of theanine (**b**), GABA (**c**), L-DOPA (**d**), dopamine (DA) (**e**) and norepinephrine (NE) (**f**) of control and theanine-administered after 30, 60 and 90 min., respectively. The obtained image data is presented using a rainbow scale and normalized versus total ion count (bottom-right bar chart); OB: olfactory bulb, AON: anterior olfactory nucleus, BS: brain stem, CB: cerebellum, CPu: caudate nucleus putamen, NAcc: nucleus accmbens, PFC: prefrontal cortex, TH: thalamus, HY: hypothalamus, HF: hippocampal formation, LV: lateral ventricle, LC, locus coeruleus, SNc: substantia nigra pars copmpacta, VTA; ventral tegmental area, MB: midbrain. n = 3. Representative IMS images are shown from one sample per group. Quantitative comparisons across groups are based on three biological replicates (n = 3), and averaged intensity values are reported separately in Supporting Information I.

Optical imaging of sagittal brain section was performed to visualize the entire mouse brain in control (non-administration LT) and at 30, 60, and 90 min following LT administration (Fig. 4a). The peak intensity values of the spectra were normalized by dividing them to the total ion current (TIC) which allows semi-quantitative comparisons between LT-administered and control mice (see supporting information I).

### LT localization

In the control sample, LT, an exogenous molecule, was neither detected nor visualized (Fig. 4b first panel from the top). In LT-administered mice, LT (*m/z* 279.1) was predominantly localized to perivascular regions surrounding the brain at 30 min post-administration, suggesting its association with blood vessels (Fig. 4b second panels from the top). Over time, LT levels in these peripheral regions gradually declined, but their presence in inner (core) brain regions increased. By 90 min, LT was largely localized in the lateral ventricle (LV) region (Fig. 4b, third and fourth panels from the top). The relative total ion counts for LT in the entire brain revealed ratios of 1:0.7:0.5 for mice administered with LT at 30, 60, and 90 min, respectively. In the control mouse brain, no LT was detected in the IMS data.

LT is one of the few amino acids capable of crossing the BBB and exerting effects in the brain via amino acid transporters^10^. LT, an amino acid structurally analogous to glutamine, is hypothesized to utilize amino acid transporters, such as glutamine transporters or neutral amino acid transporters, for its passage across the BBB^33^. Following intestinal absorption, LT enters the bloodstream and is delivered to various organs, including the brain and kidney. Unno T. et al. reported that LT is partially hydrolyzed into glutamate and ethylamine in the kidney^9^, but this does not imply that all LT undergoes hydrolysis before reaching the brain. These findings suggest that when LT is administered at sufficiently high concentrations, a portion remains intact and successfully crosses the BBB.

The observed accumulation of LT in perivascular regions suggests initial entry via the bloodstream and association with cerebral vasculature. Based on its progressive distribution, we propose that LT may access the cerebrospinal fluid (CSF) and diffuse through the ventricular system. This potential mechanism could underlie its widespread brain distribution. Further studies, such as CSF sampling or tracer-based analysis, are needed to verify this route.

### GABA dynamics in response to LT administration

In the control, γ-aminobutyric acid (GABA) exhibited limited localization in the surrounding olfactory bulb (OB), with prominent distribution observed in the prefrontal cortex (PFC) (Fig. 4c, first panel from the top). Following the LT administration, GABA levels steadily increased in the frontal lobe (Fig. 4c, second to fourth panels from the top). The relative comparison of total ion counts (TIC) for GABA in the entire brain revealed ratios of 1:2.1:1.9:4.2 for the control group (no LT administered) and mice administered LT after 30, 60, and 90 min, respectively. This increase was observed not just in the frontal regions, such as the PFC, OB, and anterior olfactory nucleus (AON), but also in the Str and thalamus (TH). By 90 min post-administration, GABA was broadly distributed throughout the brain, with prominent localization in the PFC, OB, and Str. In these locations, GABA levels increased by approximately 2.2-fold those in the control. These results are consistent with the PFC’s known role in emotion regulation, decision-making, and attention. The OB and AON are engaged in olfactory processing and have direct connections to brain regions responsible for emotional and memory activities, such as the amygdala and hippocampus. Given that mice are nocturnal and rely mainly on olfactory cues rather than visual inputs, the elevated GABA levels in these regions after LT administration indicate that LT may promote relaxation and drowsiness (sedation). There was also a noticeable progressive increase in GABA levels in the CB. The CB is vital for motor control, coordination, and balance maintenance. Consistent with these observations, mice’s activity levels dropped after LT administration (Fig. 1).

Kimura et al. previously observed that GABA levels in mice’s brains increased significantly 30 min after intraperitoneal administration of LT compared to baseline levels^34^. Kakuda et al. also found that once LT is transported into the brain, it modulates glutamine transporters and reduces glutamate supply, thereby mitigating overexcitation^35^. Glutamate, an excitatory neurotransmitter, regulates the hypothalamic–pituitary–adrenal (HPA) axis by activating glutamate receptors in the paraventricular nucleus (PVN) of the hypothalamus (HY)^36^. In contrast, GABA inhibits the secretion of corticotropin-releasing hormone (CRH), which stimulates the production of stress hormones such as adrenocorticotropic hormone (ACTH) and cortisol by activating PVN neurons^15^. We hypothesize that LT enhances GABAergic activity in the brain through two potential mechanisms. First, LT might interact with glutamine transporters^14^, possibly reducing the availability of glutamine for glutamate synthesis and thereby attenuating excitatory neuro transmission^37,38^. Second, LT might be partially hydrolyzed into glutamate, which could be subsequently converted to GABA via glutamate decarboxylase (GAD), contributing to be the observed GABA levels. These mechanisms are consistent with the increased GABA observed in emotion- and olfaction-related regions such as the PFC, OB, and TH, and may underlie the relaxation and sedation effects observed following LT administration^39,40^. In this study, GABA levels in the TH and HY increased as early as 30 min after LT administration and remained elevated at 60 and 90 min post-administration (see TH and HY regions of Fig. 4c). These findings suggest that LT may influence brain activity through GABAergic pathways.

### L-DOPA

L-DOPA was localized in the BS in both control and LT-treated mice (Fig. 4d). In control brain, the highest intensity of L-DOPA was observed in the BS. The relative comparison of total ion counts for L-DOPA in the BS indicated ratios of 1:0.9:0.6:0.56 for the control group and mice administered with LT after 30, 60, and 90 min, respectively. In contrast, in LT-administered mice, L-DOPA levels decreased in the BS with time, most likely due to its transport to the substantia nigra pars compacta (SNc) and ventral tegmental area (VTA) for dopamine (DA) production. Actually, DA increased at Str after LT administration (Fig. 4e) which coincided with a previous study^10^.

### DA

Dopamine (DA) was predominantly localized in the Str, which is divided into the caudate-putamen (CPu) and nucleus accumbens (NAcc), as observed in all samples (Fig. 4e). In the control group, DA was localized in the Str. Following the LT administration for 30 min, DA levels immediately increased by 1.6-fold and steadily increased by 1.8- and 2.0-fold in mice after 60 and 90 min, respectively, compared to the control. Notably, after 30 min, DA levels increased not only in the Str, but also in the adjacent SNc, VTA, and LC regions (Fig. 4e, second to fourth panels from the top).

DA levels in the Str peaked at 90 min after LT administration, compared to previous time points. This corresponding temporal increase in DA is consistent with the observed drop in L-DOPA levels, indicating that L-DOPA serves as a precursor to DA in the catecholamine biosynthesis pathway. DA is known to be produced in the SNc and VTA, which project to the CPu and NAcc, respectively. At 60 min after LT administration, DA production at the SNc and VTA and projection to Str peaked. At 90 min post-administration, DA storage in the Str reached its maximum level (Fig. 4e, second and third panel from the top). IMS data demonstrated that DA was preferentially localized in the CPu relative to NAcc. Relative total ion counts for DA in the CPu and NAcc yielded ratios of 1.5:1 (Fig. 4e, fourth panel from the top). This finding implies that LT enhances DA production primarily at the SNc, rather than the VTA. Although IMS provides semi-quantitative data, the preferential localization of DA in the CPu suggests enhanced production at the SNc. These observations highlight the potential role of LT in modulating DA levels, likely through its influence on biochemical pathways involved in DA synthesis, particularly in the SNc. However, further investigation is needed to elucidate the precise underlying mechanisms.

### Norepinephrine (NE)

In all samples, norepinephrine (NE) was localized in the LC and TH (Fig. 4f, top panel). At 30 min post-administration, there were no significant differences in the relative abundance or localization of NE compared to the control group (Fig. 4f, second panel from the top). By 60 min, NE levels showed a marked increase, reaching the maximum measured value. Notably, at 90 min, NE levels decreased in the LC, while they become undetectable in the TH (Fig. 4f, bottom panel). Relative total ion counts for NE in the LC and TH yielded ratios of 1:1.1:1.9:1.2 and 1:1.2:1.6:0.14, corresponding to the control group and post-LT administration at 30, 60, and 90 min, respectively.

Neurophysiologically and spatially, DA emerges as a transient intermediate in the LC before being converted into NE and projected to the TH, HY, amygdala, and other regions. Relative intensity of DA in the LC revealed ratios of 1:2.8:6.5:2.4, representing the control group and post-LT administration at 30, 60, and 90 min, respectively (Fig. 4e). Notably, NE levels increased in parallel with the rise in DA in the LC, peaking at 60 min post-administration (Fig. 4e, third panel from the top). This is consistent with the relative ratio of DA and suggests that DA produced up to 60 min contributed to NE synthesis.

NE is essential in maintaining wakefulness under normal conditions. However, excessive NE levels have been implicated in anxiety, fear, agitation, and distraction^41^. Elevated NE levels may result in increased adrenaline production which can elicit physiological symptoms such as tachycardia and cold sweats, exacerbating anxiety and agitation.

We hypothesize that the enhanced availability of DA up to 60 min after LT administration encourages the production of NE, which promotes wakefulness or alertness. This is because NE is projected to the TH in order to sustain wakefulness. However, within 90 min, the observed increase in GABA (Fig. 4c third panel from the top) may induce inhibitory control over NE synthesis in the LC, potentially contributing to a state of calmness. This further suggests that GABA plays a modulatory role in counteracting excessive NE synthesis, thereby preserving overall neurophysiological balance.

In conclusion, this study suggests that LT significantly modulates both catecholaminergic and GABAergic systems in the brain, contributing to relaxation and neurophysiological stability. IMS revealed dynamic alterations in the distribution and levels of important neurotransmitters-GABA, L-DOPA, DA and NE-across multiple brain regions following LT administration.

Behavioral analysis showed a reduction in spontaneous movement (ambulation and rearing), indicating decreased stress levels. These behavioral changes were associated with elevated GABA levels in brain regions involved in emotional regulation and sensory processing, such as the PFC, OB, and AON. DA and NE levels also showed temporally coordinated changes, suggesting that LT influences both arousal and inhibitory pathways in a region-specific manner.

Notably, increased GABA levels may be associated with changes in NE dynamics. Furthermore, the neurotransmitter profile suggests that LT may modulate neurotransmission in a manner that promotes relaxation without excessive sedation. However, further pharmacological studies are required to determine whether LT-induced GABAergic activity directly influences NE synthesis and contributes to relaxation. The ability of LT to modulate neurotransmitter dynamics supports its potential as a natural agent for lowering stress, anxiety, and improving mental clarity. Nevertheless, additional validation using depression model animals is warranted to explore its broader therapeutic potential. This study is limited by the small sample size (n = 3–4) and the use of acute time points (30–90 min), which may affect the generalizability of the findings. These findings underscore the need for futher studies using chronic LT exposure models to determine long-term neuromodulatory effects and behavioral outcomes. Future research should incorporate receptor-level analyses, including the profiling of GABAergic and adrenergic signaling pathways, and the use of pharmacological modulators to test the hypothesized interactions between GABA and norepinephrine. Such mechanistic investigations—guided by previous findings that LT modulates NE and GABA levels in vivo^42,43^—may help to clarify LT’s therapeutic potential in neuropsychiatric disorders.

## Experimental section

### Behavioral analysis

This study is reported in accordance with the ARRIVE guidelines (https://arriveguidelines.org). Eight-week-old female C57BL/6JJcl mice (23–25 g; Clea Japan, Tokyo, Japan) were used in compliance with the institutional Animal Experimental Guidelines of Fukushima University and were approved by the Laboratory Animal Care and Use Committee of Fukushima University [Permission number; B-02]. All methods were performed in accordance with relevant guidelines and regulations.

The automated motion analysis system (SCANET-40: MELQUEST Co. Ltd. Toyama, Japan) was utilized to measure spontaneous motor activity of mice. This system consists of a square cage (565 × 565 mm) with two crossing sensor frames containing of 72 (x axis) × 72 (y axis) pairs of near-infrared beam sensors, spaced 6 mm apart and at right angles to each other. The beam sensors thus form two parallel horizontal grids set at varying heights. A transparent Plexiglas cage (450 × 450 × 400 mm) for the mouse was centered within the system. Each pair of sensors was scanned every 0.1 s to detect animal movement. Two different variables of horizontal movements could be monitored by the lower sensors: small horizontal movements of 12 mm or more (M1; 1 unit = 6 mm) and large horizontal movements of 60 mm or more (M2; 1 unit = 6 mm) (see supporting information II).

For instance, when a mouse traveled 84 mm, M1 and M2 showed 14 and 1 unit, and when it traveled 48 mm, M1 and M2 showed 8 and 0 unit, respectively. The upper sensors monitored the frequency of vertical movement caused by rearing. Incomplete standing actions could be distinguished from rearing movements by adjusting the upper sensor frame. In preliminary experiments, we set both the upper and lower sensors to their lowest position: 3.75 and 8.25 cm, respectively.

To bring the mice to full alertness, they were transferred into a waiting cage under a light, 10 min before obtaining the measurement. Each mouse was then individually placed in the SCANET cage and its spontaneous locomotor activity was measured for 10 min. The locomotor scores for M1, M2, and rearing could be monitored simultaneously. In this study, the scores for M1 and M2 represent the total distances of movement and the rearing score represents the frequency of vertical movements in 10 min. The room temperature was maintained at 20 ± 2 °C. Please note that the behavioral experiments were conducted using a separate cohort of mice, independent of those used for HPLC and IMS analyses. This design was necessary because behavioral recordings were performed over a 90-min period, which is not compatible with the time-course sampling (30, 60, and 90 min) used for biochemical analyses.

### Animal experiments for High performance liquid chromatography (HPLC)-electrochemical detector (ECD) and imaging mass spectrometry (IMS)

Mice (C57BL/6JJcl male, 8W) were used. LT (200 mg/kg) or normal saline solution as control were orally administered. Separate groups of mice were sacrificed at 30, 60, and 90 min after administration by cervical dislocation. These brains were dissected and divided into the right and left brain by surgical scissors and tweezers at room temperature. The right hemisphere for quantity experiment was frozen by liquid N_2_ and stored at − 80 °C until use. The left hemisphere for IMS was embedded into a super cryo-embedding medium (Section Lab Co. Ltd., Hiroshima, Japan), flash-frozen in liquid N_2_ and stored at − 80 °C until use. In this study, the brain was manually divided into three anatomically defined regions (cerebrum, CB, and BS) for HPLC analysis. Although more refined microdissection techniques such as Palkovits’s method are available, we prioritized consistency and feasibility by adopting a uniform gross dissection protocol across all samples. This approach allowed robust comparison of neurotransmitter levels between regions within the constraints of our available instrumentation.

### HPLC-ECD

The amount of L-DOPA and dopamine was quantitated by using HPLC with ECD with an ESA coulometric detection system (Thermo Fisher Scientific, Waltham, MA, USA). The mouse right brain was divided into BS, CB and cerebrum (including Str and OB), then homogenized with a homogenizer. The resulting suspension was centrifuged to eliminate any insoluble materials. Each dissected brain region was weighed before extraction, and neurotransmitter levels were normalized to tissue weight (ng/mg) to ensure accurate and reproducible quantification.

The supernatant of rough extraction was purified by spin column (MonoSpin PBA, GL Science, USA). Apply rough extraction supernatant to spin column and centrifuge at 9,600 G for 2 min at room temperature (RT). Add 0.5 mL of 0.1 M HEPES buffer (pH 8.5) of spin column and centrifuge at 2400 G for 1 min. to remove residues. Finally, add 0.1 mL 1% acetic acid to spin column and centrifuged at 9600 G for 1 min at RT to elute target L-DOPA and dopamine from column shell.

The extracted sample was evaluated using HPLC-ECD (Thermo Fisher Scientific), which included a binary pump, degasser, autosampler, thermostated column oven and coulometric detection system (electrode detector potential at 500 mV). An ODS column (Acclaim 120C 5 µm 120 Å 4.6 × 150 mm, Thermo Fisher Scientific) was employed. A mobile phase of 20.1 M disodium hydrogen phosphate dodecahydrate, 0.05 M citric acid, 800 mg/L sodium 1-heptanesulfonate, 37.2 mg/L EDTA-2Na buffer solution-acetonitrile-methanol (1000:25.9:62.9, v/v/v) was employed at a flow rate of 1 mL/min and tun time for 35 min^44^.

### MALDI TOF–MS

Ionization of standard LT, GABA, L-DOPA, DA and NE were confirmed by MALDI-TOF-MS (rapifleX, Bruker Daltonik GmbH). The analyte surface was irradiated with 1000 laser pulses and TOF spectra were recorded in positive ion detection mode^32^ (see supporting information III).

### MALDI IMS

The left brain was cut into serial sagittal Sects. (8 μm) using a cryostat (NX70, Thermo fishier) and gently mounted on slides coated with indium tin oxide (ITO). Brain sections were applied with the TMPy solution using an artistic airbrush (Procon Boy FWA Platinum 0.2-mm caliber airbrush, Mr. Hobby, Tokyo, Japan) and subsequently incubated at 60 °C for 10 min under moisture conditions to achieve TMPy-labelled catecholamines on brain. The TMPy-labeled brain sections were sprayed with matrix (CHCA-acetonitrile/water/trifluoroaceetic acid = 70/49.9/0.1) using an automated pneumatic sprayer TM-Sprayer, HTX Tech., Chapel Hill, NC). Ten passes were sprayed with the following conditions; flow rate120 µl/min, air flow 10 psi, and nozzle speed 1100 mm/min. In order to detect the laser spot locations, the sections were scanned, and laser spot areas (200 shots) were detected with a spot-to-spot center distance (100 m) in each direction of the brain. Signals between *m/z* 100 and1000 were adjusted. The section surface was irradiated with YAG laser pulses in the positive ion detection mode. The laser power was optimized (tuned) to minimized in-source decay of targets. Obtained MS spectra were reconstructed to an image with a mass bin width of *m/z* ± 0.05 from the precise mass using FlexImaging 4.0 software (Bruker Daltonik GmbH). The peak intensity value of the spectra was normalized by dividing it with the total ion current (TIC) to achieve semi-quantitative comparisons between LT-administered and control mice. An optical image of brain section was produced using a scanner (GT-X830: Epson, Japan), followed by MALDI-TOF imaging MS of the section^31^.

### Statistical analysis

Statistical analyses were conducted using Microsoft Excel (Microsoft Corporation, Redmond, WA, USA). Welch’s t-test (two-tailed) was used for group comparisons under the assumption of unequal variances. A *p*-value < 0.05 was considered statistically significant. Given the hypothesis-driven design and small sample sizes (n = 3), multiple-comparison corrections were not initially applied. However, for additional robustness in detecting significance, False Discovery Rate (FDR) correction was subsequently performed, which did not alter the interpretation of our key results.

## Electronic supplementary material

Below is the link to the electronic supplementary material.

Supplementary Material 1

## Data Availability

All data generated or analyzed during this study are included in this published article. Additional data, including imaging mass spectrometry images and HPLC quantification data, are available from the corresponding author upon reasonable request.
