# Supplementary material for: Theanine modulation of monoamine metabolism visualized by derivatized imaging mass spectrometry
Source: Sci Rep. 2025 Jul 2;15:23075. doi: 10.1038/s41598-025-08190-0 (PMC12216342; doi:10.1038/s41598-025-08190-0)
Supplement: Supplementary file 1 — Supplementary Material 1 [file 41598_2025_8190_MOESM1_ESM.pdf]

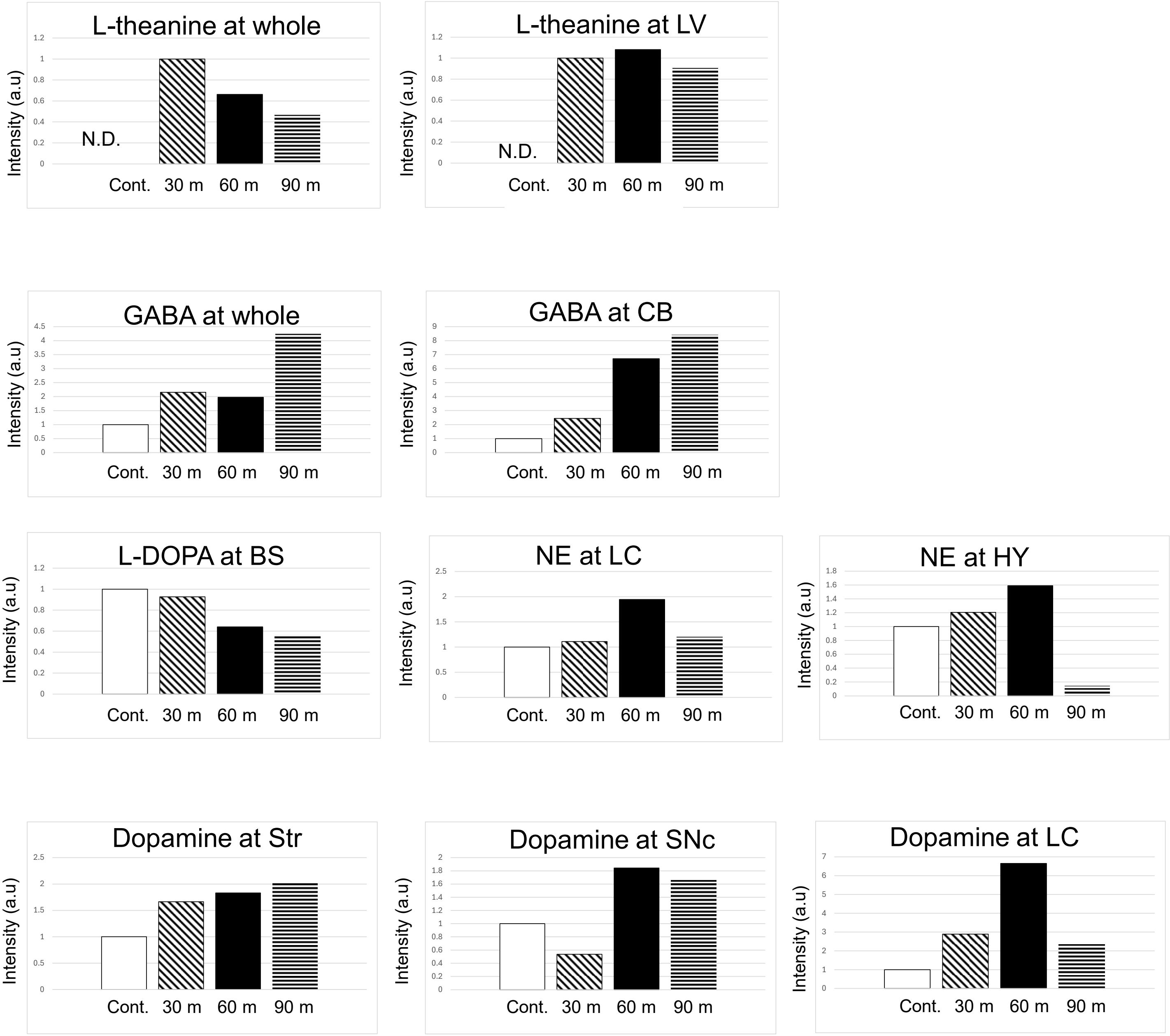

Supporting information I
Relative comparison of the theanine, GABA, L-DOPA, DA and NE in control group and post-theanine administration at 30, 60, and 90 minutes, respectively. N.D.=not detected.
Intensity (a.u.)” refers to the normalized signal intensity obtained from derivatized imaging mass spectrometry (IMS) data. Each value represents the total ion count (TIC)-normalized peak intensity for the specified molecule in the indicated brain region.

Supporting information II

As locomotor data were collected using fully automated sensors and software, behavioral scoring was not influenced by operator judgment, and blinding was not required. To reduce potential bias, animals were placed in the testing cage in a randomized order. Group assignment was known to the operator, but as measurements were automated, this did not impact the data acquisition process.

Supporting information III

The following TMPy-labeled target solutions were prepared: L-DOPA: 10 pmol/µL, DA: 10 pmol/µL and NE: 10 pmol/mL (Sigma-Aldrich, USA) and D_3-L-DOPA 10 pmol/mL (Taiyo Nippon Sanso Co., Japan): In a sealed 0.2 mL PCR test tube, combine 2.5 µL of sample solution with 7.5 µL of TMPy (Taiyo Nippon Sanso Co., Japan) (30 mM) (methanol/water/trimethyl amine=70/25/5, v/v as a reaction solution). The mixture was then heated at 60° C for 10 min. A 0.5 µL aliquot of formic acid was then added, and the solution was stored in a tightly sealed container at 4 ° C. A suspension containing TMPy-labeled catecholamine and CHCA (10 mg/mL) was placed on a target plate using a pipette. Laser fluence was set at 100 µJ/pulse, which is within the typical range for MALDI measurement of biological tissue.
